# Chloramphenicol inhibits eukaryotic Ser/Thr phosphatase and infection-specific cell differentiation in the rice blast fungus

**DOI:** 10.1038/s41598-019-41039-x

**Published:** 2019-06-26

**Authors:** Akihito Nozaka, Ayaka Nishiwaki, Yuka Nagashima, Shogo Endo, Misa Kuroki, Masahiro Nakajima, Megumi Narukawa, Shinji Kamisuki, Takayuki Arazoe, Hayao Taguchi, Fumio Sugawara, Takashi Kamakura

**Affiliations:** 1 Tokyo University of Science, Department of Applied Biological Science, Faculty of Science and Technology, 2641, Yamazaki, Noda, Chiba 278-8510 Japan; 2 Osaka University, Research Institute for Microbial Diseases, Department of Molecular Microbiology, 3-1 Yamadaoka, Suita, Osaka 565-0871 Japan; 3 Azabu University, Department of Veterinary Science, Laboratory of Basic Education, 1-17-71 Fuchinobe, Chuo-ku, Sagamihara-shi, Kanagawa 252-5201 Japan

**Keywords:** Chemical genetics, Microbial genetics

## Abstract

Chloramphenicol (Cm) is a broad-spectrum classic antibiotic active against prokaryotic organisms. However, Cm has severe side effects in eukaryotes of which the cause remains unknown. The plant pathogenic fungus *Magnaporthe oryzae*, which causes rice blast, forms an appressorium to infect the host cell via single-cell differentiation. Chloramphenicol specifically inhibits appressorium formation, which indicates that Cm has a novel molecular target (or targets) in the rice blast fungus. Application of the T7 phage display method inferred that MoDullard, a Ser/Thr-protein phosphatase, may be a target of Cm. In animals Dullard functions in cell differentiation and protein synthesis, but in fungi its role is poorly understood. *In vivo* and *in vitro* analyses showed that MoDullard is required for appressorium formation, and that Cm can bind to and inhibit MoDullard function. Given that human phosphatase CTDSP1 complemented the MoDullard function during appressorium formation by *M. oryzae*, CTDSP1 may be a novel molecular target of Cm in eukaryotes.

## Introduction

Drugs frequently exhibit unexpected or unintended activities, as a result of unknown targets or interaction between drugs and other host molecules. A number of marketed drugs are assumed to have multiple targets^1,2^. Although identification of new drug targets is vital for discovery and development of novel drugs, the procedure is challenging, labour intensive, and time-consuming for researchers and the pharmaceutical industry^3,4^. By use of gene microarrays, candidate genes for intracellular drug targets affected by specific drug activities have been identified^5^. However, this strategy generates a substantial amount of data and changes in gene expression may not correlate with drug action and hidden phenotypes. If target engagement is not validated, novel mechanistic targets are difficult to identify. Recent advances in validation analysis have enabled evaluation and estimation of novel targets of drugs. The use of fluorescent proteins in living cells has enabled the quantitation of spatial and temporal changes in drug-target engagement in protein complexes in response to drug treatment^6,7^. Drug–target interactions are also predicted using machine learning-based and network-based methods^8^. These powerful validation strategies accelerate identification of the drug-target engagement. In contrast, for development of new drugs together with target estimation, which integrates the relationship with known target factors, it is necessary to seek unknown target factors and to elucidate their functions.

The rice blast fungus *Magnaporthe oryzae* (anamorph *Pyricularia oryzae*) is one of the most destructive pathogenic filamentous fungi that infect rice. Rice blast decreases the total yield of crops by approximately 10–30%^9^, therefore understanding the infection strategy of this pathogen and controlling damage to crops are critical for global food security. The infection cycle of this fungus is initiated by a three-celled conidium contacting the surface of a rice leaf. The conidium forms an elongating germ tube, and subsequently a hemispherical structure, termed the appressorium, develops at the tip of the germ tube. Considerable osmotic pressure attaining approximately 8 MPa is generated in the appressorium owing to accumulation of high concentrations of compatible solutes, such as glycerol^10,11^. Using this turgor pressure, a penetration peg extends from the appressorium base and penetrates the plant cell wall to enter an epidermal cell. This infection process is necessary to invade a host plant and is dependent on mitotic division of the single-celled germ tube and cell differentiation^12^. In addition, many molecules stored in the conidium are associated with appressorium formation, and expression of numerous appressorium-related genes increases dramatically during the cell differentiation process^13,14^.

Given that innumerable cellular components are involved in cell differentiation, there are many potential molecular targets of a variety of drugs. Knowledge of drugs that inhibit cellular differentiation will contribute to understanding not only secondary targets of drugs but also the underlying cellular events. Ishii *et al*. focused on simple cell differentiation and unique genomic properties of *M. oryzae*, and reported that roxithromycin can inhibit appressorium formation via interaction with MoCDC27^15^. These authors used a *M. oryzae* appressorium assay for drug screening and a genomic DNA library-based T7 phage display method for identification of the roxithromycin target. *M. oryzae* conidial germination and appressorium formation can be artificially induced on hydrophobic polyvinyl chloride as well as on plant surfaces^16^. The genome size of *M. oryzae* is approximately 41.7 Mb and the proportion of non-coding DNA is about 60%, whereas the human genome is approximately 2.8 Gb and non-coding DNA comprises about 99% of the genome^17^. Although a genomic library includes the peptides derived from non-coding regions, frame-shifting, and antisense sequences, *M. oryzae* genomic DNA library enables comprehensive analysis of the majority of proteins encoded by temporarily expressed genes. These genomic characteristics of *M. oryzae* are suitable for seamless identification of novel drug targets in a eukaryote: screening for cell differentiation-specific drug targets, identification of the candidate target using the fungal genomic DNA library and T7 phage display method, and validation of the function of the candidate protein and its interaction with drugs.

In this study, we show that simple screening and *in vivo* validation methods using *M. oryzae* as the study organism are valuable to discover unexpected drug targets. The appressorium formation assay revealed that a classic antibiotic, chloramphenicol (Cm), specifically inhibited appressorium formation in *M. oryzae*. It is well known that Cm binds to 50 S ribosomal RNA and inhibits peptide synthesis in prokaryotes^18–20^. Although a high concentration of Cm may cause mitochondrial malfunction in eukaryotic cells via the prokaryotic ribosome^21^, the effect of Cm on appressorium formation in *M. oryzae* suggests the existence of novel secondary targets in fungi. The original genomic library-based T7 phage display method revealed that Cm can target the Ser/Thr phosphatase Dullard in *M. oryzae* and humans. Therefore, the Dullard protein may be a secondary target of Cm in humans, which has not been explored previously. This is the first report that Cm targets a eukaryotic molecule and inhibits cell differentiation. We demonstrated that fungal genomic library-mediated comprehensive screening and assay methods may contribute to identification of novel drug targets associated with cellular differentiation in eukaryotes.

## Results

### Cm specifically inhibited appressorium formation of *M. oryzae*

**Figure 1:**
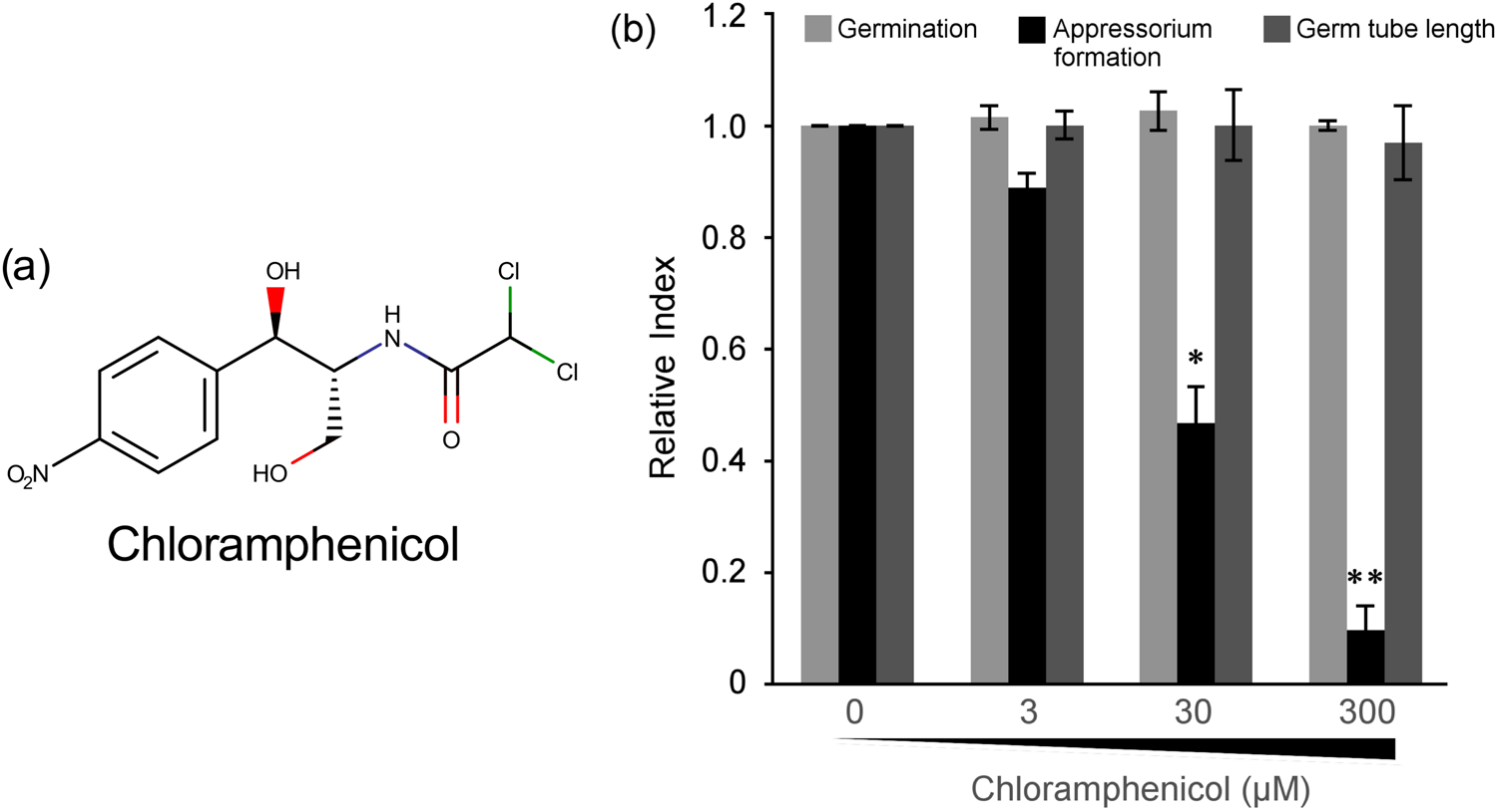
Inhibitory ability of chloramphenicol on *Magnaporthe oryzae*. (**a**) Structure of chloramphenicol (Cm). (**b**) Inhibitory effect of Cm on conidial germination, germ-tube elongation, and appressorium formation. Conidial suspensions of the wild-type *M. oryzae* P2 strain were inoculated on plastic cover slips in the presence of various concentrations of Cm diluted by 1% ethanol. The percentages of conidial germination and appressorium formation, and the length of non-appressorium-forming germ tubes were assessed on hydrophobic plastic cover slips at 6 h post inoculation. Each score was standardised against that of 0 µM Cm (control). *p < 0.05, **p < 0.01 (Student’s *t*-test) compared with 0 µM Cm. Error bars indicate the standard error. The experiment was performed in triplicate for each sample and repeated three times.

To test whether Cm affects infection-specific cell differentiation of *M. oryzae*, conidial suspensions supplemented with 3, 30, 300, or 3000 µM Cm were inoculated onto the surface of hydrophobic polyvinyl chloride. After 6 h post inoculation, the percentage of germinated conidia, germ-tube length, and percentage appressorium formation were calculated after microscopic observation. The germination percentage and germ-tube length were not affected by Cm, whereas the percentage of appressorium formation was significantly and specifically decreased in the presence of 30 and 300 µM Cm (Fig. 1). Addition of excessive Cm (3000 µM) resulted in inhibition of conidial germination and/or germ-tube elongation, which suggested that this Cm concentration inhibited the mitochondrial ribosome (Fig. S1). Interestingly, the Cm analogues thiamphenicol and florfenicol did not inhibit appressorium formation (Fig. S2). In these analogues the nitro group of Cm is modified, which suggests that the nitro group of Cm is involved in the inhibitory effect from binding to the target factor. These results indicated that Cm specifically inhibits appressorium formation of *M. oryzae* and has a novel target of cell differentiation in eukaryotic cells.

### Isolation of MoDullard as the Cm target using the T7 phage display method

**Figure 2:**
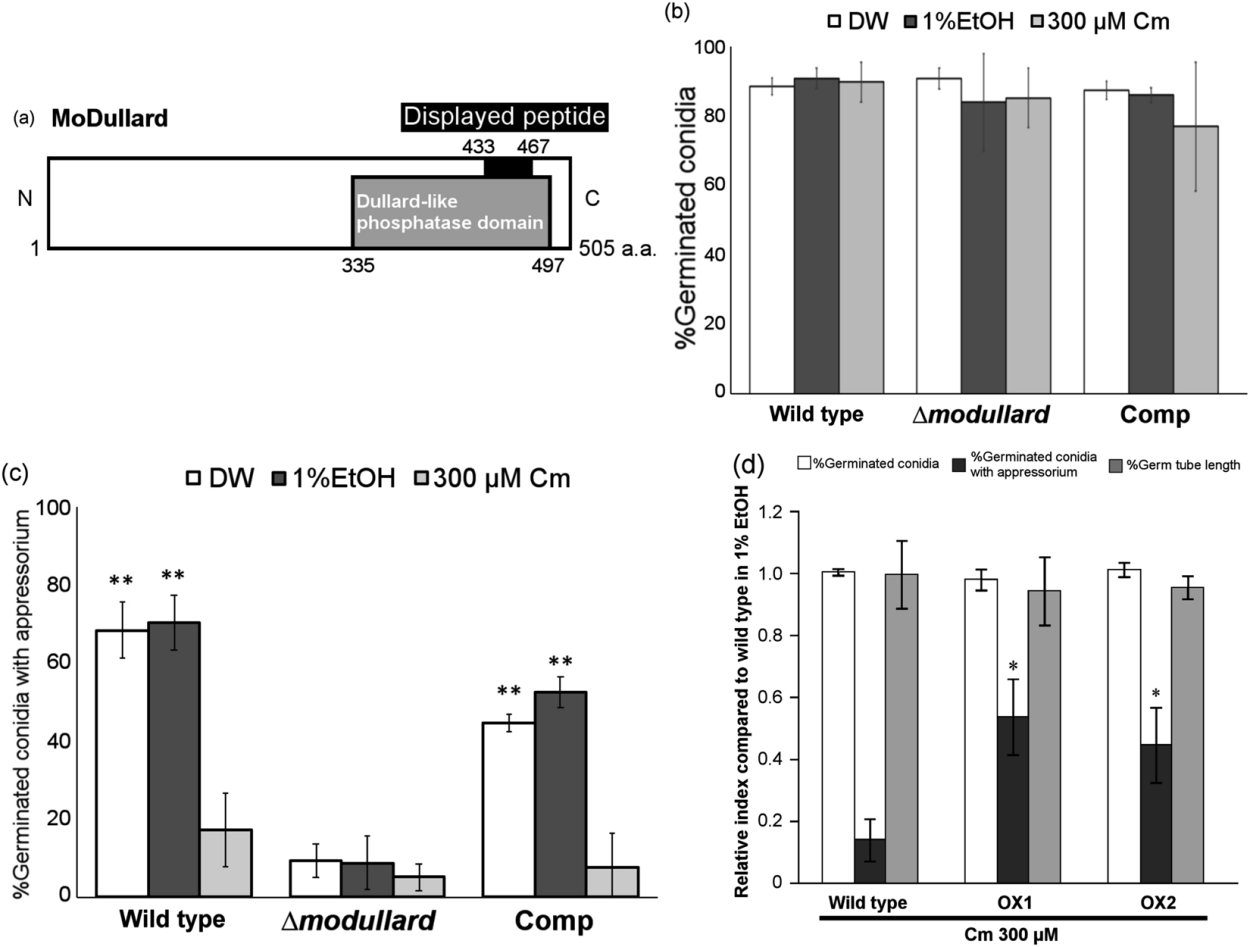
Structure and functional analysis of MoDullard. (**a**) Domain composition of MoDullard. The gray bar represents an annotated Dullard-like phosphatase domain and the black bar indicates the section that was estimated to bind to chloramphenicol (Cm) using a T7 phage display method. (**b**) Conidial germination percentage and (**c**) appressorium formation percentage in *∆modullard* and complementary strain. Each conidial suspension was treated with distilled water, 1% ethanol, or 300 µM Cm in 1% ethanol. **p < 0.01 compared with *∆modullard* (Student’s *t*-test). (**d**) Conidial germination, germ-tube length, and appressorium formation percentage in *MoDULLARD* overexpression mutants in 300 µM Cm. Each percentage was assessed at 6 h post inoculation and was standardised against that of 0 µM Cm (control). *p < 0.05 compared with wild-type (Student’s *t*-test). Error bars indicate the standard error. The experiments were performed in triplicate for each sample and repeated three times.

To screen the novel Cm target in *M. oryzae*, we used the *M. oryzae* genomic DNA library-based T7 phage display method^15,22^. As the ligand for T7 phage display, we connected Cm to a biotinyl linker (Bio-Cm) by organic synthesis (Fig. S3). Biotinylated Cm retained the ability to inhibit appressorium formation of *M. oryzae* (Fig. S4). Using this approach, we obtained 82 candidate peptide sequences, of which a BLASTP search revealed that 14 sequences showed homology to *M. oryzae* proteins. Among these sequences, two were coded in CDS regions (Table S2) and one of the candidate peptides showed high similarity (e-value: 1.6 × 10^−9^) to the Dullard-like phosphatase domain in the Ser/Thr phosphatase Dullard (MGG_03646; MoDullard) (Fig. 2a). Dullard was identified in *Xenopus laevis* and Dullard-like phosphatase is highly conserved in eukaryotes^23^. Orthologues in ascomycetes and yeast have been identified, and the phosphatase domain is also conserved between these organisms (Fig. S5). Dullard is involved in cell differentiation in higher organisms; for example, Dullard acts as a negative regulator of TGF-β signaling for endochondral ossification via phosphorylation of Smad2/3 in mice^24,25^. *M. oryzae* harbored two paralogues of *MoDULLARD*, namely *MoNEM1* (nuclear envelope morphology protein 1) (MGG_06001) and *MoFCP1* (RNA polymerase II subunit A domain phosphatase 1) (MGG_03485). These genes also contained a putative Dullard-like phosphatase domain. Although pathogenicity was not affected in the deletion mutant *monem1* in barley and rice^26^, a functional analysis of MoDullard and MoFCP1 in *M. oryzae* has not been performed. Given that MoDullard contained the completely identical sequence to the displayed peptide sequence, we performed a functional analysis of MoDullard.

### MoDullard is associated with appressorium formation

To examine whether Dullard phosphatase is associated with appressorium formation by *M. oryzae*, we analysed the expression patterns of MoDullard by semi-quantitative RT-PCR (semi-qPCR). *MoDULLARD* was expressed during vegetative growth and appressorium formation but a higher expression level was observed in the appressorium formation phase (Fig. S6a). To investigate the effect of MoDullard on appressorium formation, we generated the *Δmodullard* mutant by *Agrobacterium tumefaciens*-mediated transformation (AtMT)^27^. The mutant often produced aberrant conidia that were narrow and/or lacking one of the two septa (Fig. S6b), and showed a slightly reduced growth rate on oatmeal medium, YG medium, and YPD medium (data not shown). The conidia from the *∆modullard* mutant and the wild-type P2 strain showed similar germination frequencies (Fig. 2b), whereas the percentage appressorium formation of the *Δmodullard* mutant was severely decreased compared with that of the wild type (Fig. 2c). The *MoDULLARD*-complemented strain showed restoration of appressorium formation and susceptibility to Cm. In addition, the *MoDULLARD* overexpression strain showed tolerance to Cm (Fig. 2d). These results indicated that MoDullard plays an important role in appressorium formation and that Cm may directly bind to MoDullard. To analyse the interaction between MoDullard and Cm, the GST-tag and 6xHis-tag were fused to the N-terminus and C-terminus of MoDullard, respectively. The recombinant MoDullard and control proteins were injected into avidin-beads to immobilise Bio-Cm or Bio-Ctrl. The bound proteins were eluted and detected by western blotting. The tag-fused recombinant MoDullard protein bound weakly to Bio-Cm (Fig. S7). From these inhibition analyses, it was inferred that Cm inhibits appressorium formation by means of the loss or decline in Ser/Thr phosphatase function.

### MoDullard function is similar to that of human CTDSP1

**Figure 3:**
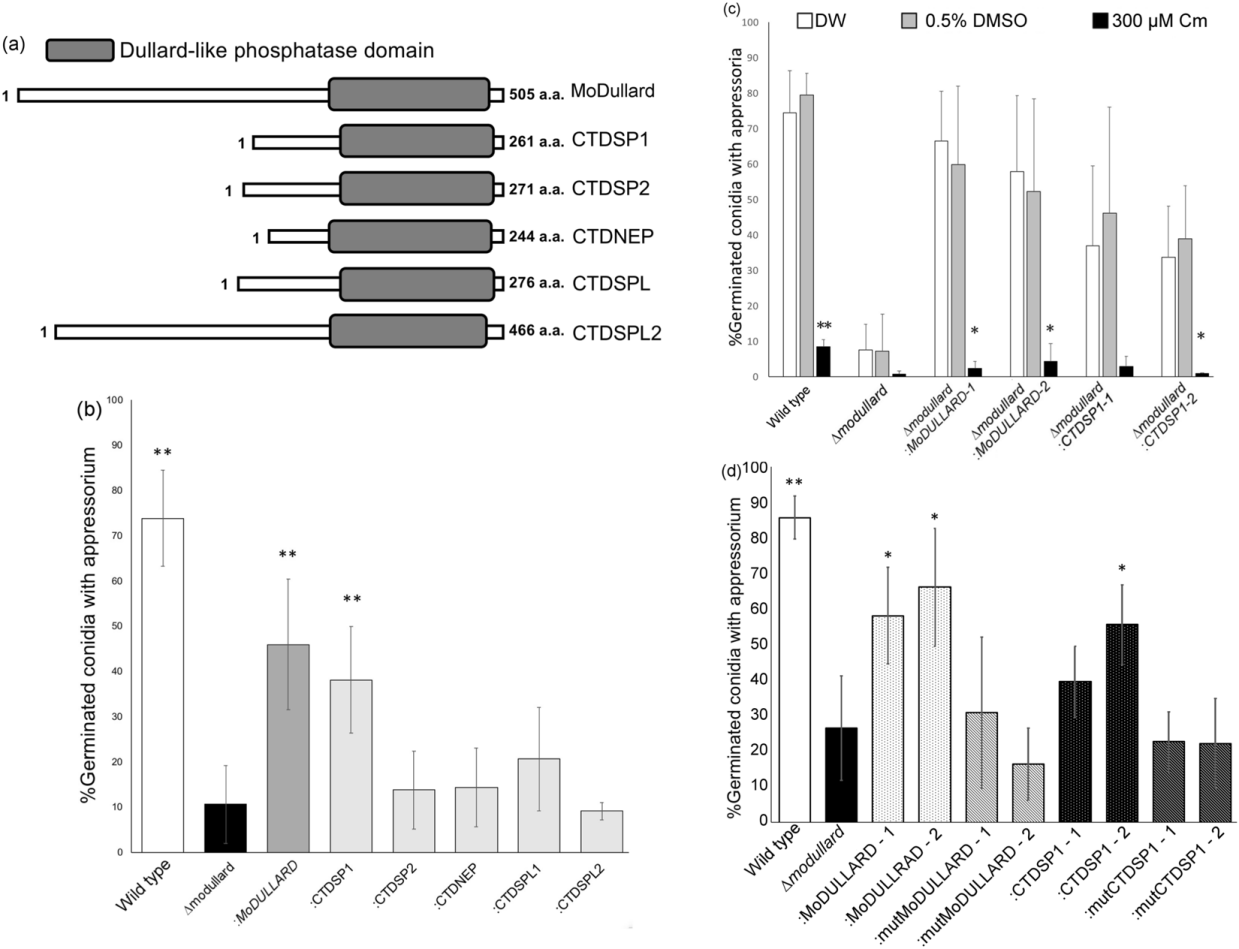
Analysis of MoDullard orthologues in humans. (**a**) The domain composition of MoDullard homologues in humans: CTDSP1 (NP_067021.1), CTDSP2 (NP_005721.3), CTDNEP (NP_056158.2), CTDSPL (NP_001008393.1), and CTDSPL2 (NP_057480.2). The gray box indicates each Dullard-like phosphatase domain contained at the carboxyl terminus. (**b**) Appressorium formation percentage in human homologue complementary strains. Each conidial suspension was treated with distilled water. (**c**) Inhibitory effect of chloramphenicol (Cm) on CTDSP1 complementary strains. Each conidial suspension was treated with distilled water, 0.5% DMSO, or 300 µM Cm in 0.5% DMSO. *p < 0.05, **p < 0.01 compared with 0.5% DMSO (Student’s *t*-test). (**d**) Appressorium formation percentage of complementary strains. DxDxT/V motifs of mutMoDULLARD and mutCTDSP1 were inactivated. The appressorium formation percentage was assessed at 6 h post inoculation. *p < 0.05, **p < 0.01 compared with *∆modullard* (Student’s *t*-test). Error bars indicate the standard error. The experiments were performed in triplicate for each sample and repeated three times.

Chloramphenicol causes side effects in humans but its target molecule has not been elucidated in eukaryotes. Given that fungal Dullard was targeted by Cm, we investigated whether human orthologues complement the MoDullard function in *M. oryzae*. Five MoDullard orthologues, namely CTDSP1 (CCDS56166.1), CTDSP2 (CCDS41801.1), CTDNEP (CCDS11093.1), CTDSPL (CCDS33734.1), and CTDSPL2 (CCDS10110.1), were identified from the human genome (Fig. 3a). Each orthologue also contained a phosphatase domain in the C-terminal region with high similarity (Fig. S8), but these orthologues have different molecular functions in human cells^28–32^. We cloned cDNA for each orthologue from human U937 cell mRNA and expressed the cDNA under the constitutive promoter in *Δmodullard* cells. The appressorium formation assay surprisingly showed that only *CTDSP1* expression complemented the MoDullard function (Figs 3b, S9) and Cm inhibited *CTDSP1*-complemented appressorium formation (Fig. 3c). To confirm the importance of phosphatase activity for appressorium formation, we obtained the complementary strains expressing the active-site-mutated MoDullard and CTDSP1 in the *Δmodullard* mutant, and the appressorium formation ability of these mutants was analysed. All complemented strains containing the mutated phosphatases could not restore appressorium formation (Fig. 3d). These results suggested that MoDullard affected phosphorylation of the carboxy-terminal domain (CTD) in RNA polymerase II (RNAPII) as well as CTDSP1.

## Discussion

Organisms are composed of many thousands of compounds, thus many drugs may have multiple molecular targets and cause side effects. Analysis of secondary targets of drugs will lead to drug repositioning or analysis of the mechanism of the side effects^1^. Chloramphenicol was isolated from *Streptomyces venezuelae* as a broad-spectrum antibiotic for prokaryotes but often causes side effects in humans; therefore, use of Cm has been avoided as a first-line treatment^33^. In the present study, Cm showed specific inhibition of appressorium formation in *M. oryzae*, although conidial germination and germ-tube elongation were not affected. This effect cannot be explained by the well-known action of Cm binding to the mitochondrial ribosome. These results suggested that Cm may target a novel eukaryotic molecular factor (or factors) and inhibit cell division and differentiation factors in the rice blast fungus.

Complementation experiments indicated that MoDullard and the human orthologue CTDSP1 fully complemented the function of MoDullard in *M. oryzae*. This is the first report that MoDullard and human CTDSP1 play an important role in cell differentiation in filamentous fungi and are targeted by Cm in eukaryotic cells. MoDullard contains the highly conserved Dullard phosphatase domain and is a member of the halo acid dehalogenase (HAD) superfamily, which is classified in the RNA polymerase CTD phosphatase family^34^. In human cells, CTDSP1 functions as a Ser/Thr phosphatase, especially in the dephosphorylation of the CTD of the RNAPII largest subunit (Rbp1)^35^. The human RNAPII CTD contains highly conserved heptad repeats Tyr-Ser-Pro-Thr-Ser-Pro-Ser (YSPTSPS)^36^. After RNAPII-mediated transcription, dephosphorylation of the CTD heptad repeats of RNAPII released from the DNA template is required to form the complex for the next transcription cycle^37^. Therefore, the phosphorylation status of the CTD repeat regulates RNAPII-mediated transcription, and dephosphorylation of repeats is needed to recycle RNAPII. A mutation in the phosphatase domain causes loss of the dephosphorylation activity of CTDSP1^38^. Human CTDSP1 complemented the MoDullard function in *M. oryzae* cells, and the function was inhibited by treatment with Cm during appressorium formation. In addition, mutation of the phosphatase domain in this protein led to a severe decrease in frequency of appressorium formation. These results suggested that MoDullard also plays a role as a Ser/Thr phosphatase for dephosphorylation of the CTD of the RNAPII largest subunit Rbp1. The abundance of total RNAPII cannot be changed but raising the rate of RNAPII recycling improves the transcription efficiency^39^. RNAPII is phosphorylated stepwise during initiation, accumulation of RNA, and release of the expression products. The stepwise phosphorylation is the key to start each step and at the termination RNAPII is hyperphosphorylated; RNAPII is dephosphorylated to enter the next transcriptional stage^35^. Consequently, the phosphorylated RNAPII must be dephosphorylated to restart the transcription cycle. During infection of a rice plant by *M. oryzae*, energy resources are limited to those stored in the conidium. Thus, energy management is important for successful infection under the energy-limited condition. Conidial autophagic cell death occurs and subsequently the substrates and energy are recycled for appressorium formation^40^. *M. oryzae* also prepares and exports various effectors into the host cells to antagonise plant immunity, such as the pathogen-associated molecular patterns-triggered defence response, effector-triggered immunity, and the hypersensitive response^41,42^. Therefore, dephosphorylation of RNAPII–CTD to recycle RNAPII is important in pathogenesis, and the transcription cycle would be delayed by loss-of-function of the Dullard protein. Excluding the treatment with a high concentration of Cm (3000 µM), Cm did not inhibit conidial germination and germ-tube elongation (Fig. S1). If MoDullard functions similar to CTDSP1 in the dephosphorylation of RNAPII, Cm may affect the recycling of RNAPII via phosphatase activity during appressorium formation. Previous studies suggest that dormant conidia of ascomycetes store a pre-existing pool of mRNAs and ribosomes for immediate use in conidial germination and germ-tube elongation^43,44^. We infer that the pre-existing mRNA may be used for conidial germination and germ-tube elongation in *M. oryzae*, which overrides the inhibition of phosphatase and RNA synthesis by Cm treatment.

Human CTDSP1 interacts with CdcA3 (Cell division cycle associated 3), MBP (Myelin basic protein)^45^, REST (RE1-silencing transcription factor)^28^, SNAI1 (snail family zinc finger 1)^46^, and RNAPII–CTD^47^. In the *M. oryzae* genome, MoCos1 (Conidiophore stalk-less1, MGG_03977) showed high amino acid sequence homology to SNAI1 (E-value: 7.2 × 10^−15^) and REST (RE-1 silencing transcription factor; 1.1 × 10^−11^). Mst12 (Transcription factor SteA, MGG_12958) also showed high homology to SNAI1 (6.3 × 10^−13^) and REST (2.7 × 10^−6^), but homologues of Cdc3A and MBP were not identified. MoCos1 and MoSteA, which are His_2_-Cys_2_ zinc finger proteins, may be the cofactors of MoDullard in *M. oryzae*. However, the *∆mst12* mutant is capable of appressorium formation, although infection of the host cell by this mutant fails owing to defective penetration^48^. *M. oryzae* produces a cascade of Osm1, MoMsn1, and MoCos1, but the *∆osm1* mutant also forms a normal appressorium^49^. Given that the *∆momsn1* mutant is unable to produce conidia, the relationship between the function of MoMsn1 and appressorium formation has not been determined^49^. In *Saccharomyces cerevisiae*, Psr1p, the orthologue of MoDullard, co-functions with Whi2p and is involved in the sodium stress response^50,51^. The proteins Psr1 and Whi2 of *Colletotrichum orbiculare*, a plant pathogenic fungus that infects melons and cucumber, are associated with the target of rapamaycin (TOR) pathway and regulate pleiotropic cellular signalling^52^. The TOR pathway is broadly conserved among eukaryotes and regulates cell growth and proliferation in response to nutrients^53^. The *∆copsr1* mutant shows extension of infection hyphae following appressorium formation, but the role of Psr1 in appressorium formation is poorly studied in filamentous fungi. The tag-fused recombinant MoDullard protein bound weakly to Bio-Cm (Fig. S7). We thought that the binding of Cm to MoDullard may require cofactors in the cell. From these findings, we speculate that there are other cofactors of MoDullard and mechanisms for the dephosphorylation of RNAPII–CTD and appressorium formation in *M. oryzae*.

MoDullard orthologues, CTDSP1, CTDSP2, CTDNEP, CTDSPL, CTDSPL2, show a different function and are associated with cellular differentiation in human cells^28–32^. From the sequence similarity of the displayed peptide, no remarkable differences were observed among MoDullard, CTDSP1, and the other homologues at the Cm binding site of the Dullard-like phosphatase domain. These sequence similarities raise the possibility that Cm can bind not only MoDullard and CTDSP1 but also the domain of the homologues. However, functional restoration of appressorium formation was not observed in each transformant with these homologues. Therefore, it is unknown whether Cm shows the ability to bind to these homologues nor whether the homologues play a functional role in appressorium formation.

In this study, we obtained novel insights into cell differentiation in *M. oryzae* and a secondary target of Cm in eukaryotic cells by using a simple cell differentiation-based unique screening method. We infer that this strategy will contribute to understanding eukaryotic cell differentiation and drug secondary targets.

## Materials and Methods

### Fungal strains and growth conditions

*M. oryzae* P2 strain, a Japanese rice blast pathogenic isolate, was used as the wild-type strain in this study. For preservation, P2 and derived strains were cultured on oatmeal agar medium containing 5.0% oatmeal (Quaker Oats Company, Chicago, IL, USA), sucrose (Nacalai Tesque, Kyoto, Japan), and 1.5% agar (FUJIFILM Wako Pure Chemical Corporation, Osaka, Japan) at 28 °C. For induction of conidiation, aerial hyphae were removed with a sterilised brush and stationary-cultured under blacklight blue lamps (FL20S, 20 W; Toshiba Co. Ltd, Tokyo, Japan) at 28 °C for 2–3 days. Conidia were brushed off into sterile water and used in subsequent experiments.

### Appressorium formation assay

The percentages of germinated conidia and appressorium formation were determined by means of an appressorium formation assay as described previously^54^. Chloramphenicol (Wako) was dissolved in ethanol (Nacalai Tesque) or dimethyl sulfoxide (DMSO; Wako) and a stock solution (10 mg ml^−1^) was prepared. The stock solutions were diluted to the appropriate concentrations with each solvent. Before placing drops of conidial suspension on the surface of hydrophobic polyvinyl chloride (Thermo Fisher Scientific, Inc., Waltham, MA, USA), diluted Cm solution was mixed with conidial suspensions at various concentrations in 0.1% ethanol or 0.5% DMSO.

### Phage display method

For screening we used the T7Select^®^ Phage Display System (Novagen, Madison, WI, USA) following the manufacturer’s protocol. For the T7 phage display method, a *M. oryzae* genomic DNA library was used, which was constructed as previously reported^15^. As bait, Bio-Cm was immobilised on 5 µg NeutrAvidin™ Protein (Pierce, Rockford, IL, USA) placed on the sensor chip of an AFFINIX Q QCM apparatus (Initium, Kanagawa, Japan). Non-immobilised avidin was removed using an air duster (Sanwa Supply, Tokyo, Japan). Bio-Cm dissolved in 20 µl QCM buffer (10 mM Tris-Cl, 200 mM NaCl; Nacalai Tesque) with 10% DMSO was placed on the avidin-immobilised chip and left at room temperature for 80 min. Non-immobilised Bio-Cm was removed using an air duster. The chip was set up for the QCM apparatus with the cuvette containing 8 ml QCM buffer. An aliquot (80 µl) of the T7 phage display library was injected into the cuvette. The frequency changes, which were caused by binding between phages and Bio-Cm on the sensor chip, were monitored for 10 min. The chip was detached from the apparatus and air-dried, then bound phages were recovered by applying a 10 µl drop of host *Escherichia coli* (BLT5615). The isolated phage DNA sequence was amplified by PCR and sequenced.

### Extraction of DNA and RNA from *M. oryzae*

For induction of hyphal growth to extract genomic DNA, *M. oryzae* strains were cultured in 20 ml YG liquid medium containing 0.5% yeast extract and 2% glucose (Nacalai Tesque) and incubated at 28 °C on a rotary shaker at 150 rpm for 2 days. The fungal mycelium samples were inoculated into 100 ml YG liquid medium and incubated at 28 °C on a rotary shaker at 150 rpm for 1 day. The fungal fluid was concentrated by centrifugation at 2000 × *g* at room temperature for 10 min. After freeze-fracturing with liquid nitrogen, genomic DNA was extracted using a previously described method^15^. The extraction of RNA at the conidial germling stage was performed following a previously described method^54^.

### Construction of MoDullard deletion mutant

To establish a *Δmodullard* strain knockout vector using the AtMT method, we constructed pNR011 as the AtMT knockout vector. Restriction enzymes and calf intestinal alkaline phosphatase (New England Biolabs Inc., Ipswich, MA, USA), Ligation Convenience Kit (Wako), and QIAquick^®^ Gel Extraction Kit (QIAGEN, Hilden, Germany) were used for vector construction. First, the vectors pBI121 and pRI910 were digested using *Pme*I and *Eco*RI. The extracted pRi replicator region (9265 bp) from pRI910 and multiple cloning site from pBI121 were ligated into the vector pNR01. The AtMT knockout vector, pNR011, was constructed by cloning PtrpC-*HPH* from pCSN43^55^ into pNR01 using *Sma*I. The upstream and downstream regions of *MoDULLARD* were amplified from genomic DNA using the primers MoDul-Up-Fwd-KpnI, MoDul-Up-Rvs-KpnI, MoDul-Down-Fwd-XbaI, and MoDul-Down-Rvs-XbaI, and each region was cloned into pNR011 digested with *Kpn*I or *Xba*I. The cloned vectors were digested with *Rsr*II, and several fragments containing the upstream or downstream region were ligated to construct the *modullard* knockout vector pANKO03.

To obtain *Δmodullard* mutants, the AtMT method was performed using a previously reported procedure^27^. After co-culture fungal and bacterial cells on paper filters were plated on YG agar medium supplemented with hygromycin (500 µg ml^−1^; Nacalai Tesque) and Merpenem (25 µg ml^−1^; Nacalai Tesque) at 28 °C for 4 days. Individual transformants that emerged at the edge of the paper filters were transferred onto oatmeal medium and incubated for 4 days. The deletion of *modullard* in the transformants was checked by PCR and Southern blot analysis.

### Cloning and construction of plasmid vectors for transformation of *M. oryzae*

To express each gene in *∆modullard*, we constructed the pBFT vector by cloning the promotor of the translation elongation factor gene (Ptef) fragment from pMK412^56^, amplified using the primers pTEF-Fwd-PmeI and pTEF-Rvs-BamHI, and digested with *Pme*I and *Bam*HI. The pBF101 vector^57^ was also digested by *Pme*I and *Sma*I, then ligated with the digested PCR fragment to generate pBFT. To construct each expression vector, corresponding genes were amplified with the primers MoDUL-Fwd, MoDUL-Rvs, CTDSP1-Fwd, CTDSP1-Rvs, CTDSP2-Fwd, CTDSP2-Rvs, CTDNEP-Fwd, CTDNEP-Rvs, CTDSPL-Fwd, CTDSPL-Rvs, CTDSPL2-Fwd, and CTDSPL2-Rvs from *M. oryzae* cDNA (extracted from germling-stage conidia) or human cDNA (extracted from U937 cultured cells using RNAzol; Cosmo Bio Co., Ltd., Tokyo, Japan) following the manufacturer’s protocol. The amplified DNA fragments were digested by *Spe*I or *Bam*HI, then cloned into pBFT to construct each expression vector. The sequence in each vector was checked by sequencing.

To obtain complementary strains harboring the inactivated DxDxT/V motif, the mutation induction vectors were constructed using improved methods for site-directed mutagenesis using the Gibson Assembly^®^ Master Mix (New England Biolabs) following the manufacturer’s protocol. Using the primers mutDxDxT-Fwd and mutDxDxT-Rvs, the sequence of the DxDxT/V motif in the expression vectors was changed from 5′-GATCTCGACGAGACG-3′ to 5′-aATCTCaACGAGACG-3′ to mutate DxDxT to NxNxT (lower-case letters indicate the nucleotides changed from the original sequence). The sequences of the constructed vectors were checked by sequencing.

### Expression and purification of proteins

To obtain purified proteins for the binding assay, we used pGEX-6p-1 (GE Healthcare UK Ltd, Amersham, UK) as an expression vector. The cDNA of interest was amplified from complementary vectors by MoD-EX-Fwd-XhoI and MoD-EX-Rvs-BamHI. Amplified fragments were digested using *Xho*I or *Bam*HI and were cloned into pGEX-6p-1. The sequence of the vectors and that each cDNA was in-frame was checked by sequencing. The obtained vector was introduced to Rosetta™(DE3) Competent Cells (Merck KGaA, Darmstadt, Germany) and used as an expression host. For the expression experiment Auto Induction medium^58^ was used, which contains autoclaved A solution (final concentration 2.4% yeast extract and 1.2% Trypton; Nacalai Tesque), autoclaved B solution (1.1% KH_2_PO_4_, Nacalai Tesque; and 4.7% K_2_HPO_4_, Kanto Chemical Co., Tokyo, Japan), and filtered sugar solution (0.6% glycerol, 0.5% glucose, and 0.08% lactose; Nacalai Tesque). The expression hosts were harvested on LB agar medium supplemented with carbenicillin (25 µg ml^−1^) and the single colony was transferred to 1 ml LB liquid medium supplemented with carbenicillin (25 µg ml^−1^) and incubated at 37 °C overnight. An aliquot (100 µl) of the bacterial suspension was inoculated in 6 ml LB liquid medium supplemented with carbenicillin (25 µg ml^−1^) and incubated at 37 °C overnight. The pre-cultured suspension was injected into Auto Induction medium supplemented with carbenicillin (25 µg ml^−1^) to OD_600_ of about 0.5 and incubated at 37 °C for 24 h. The incubated bacterial suspension was collected by centrifugation at 5000 × *g* for 5 min, and the pellet of *E. coli* cells was washed twice with 1 ml TBS buffer. The washed pellet was suspended in 1 ml sonication buffer containing Tris-Cl (pH 8.0), 150 mM NaCl, and 1 mM ethylenediamine-*N*,*N*,*N*,*N*′,-tetraacetic acid disodium salt, dehydrate (Wako). Before sonication, 1 mM phenylmethylsulfonyl fluoride (Nacalai Tesque) dissolved in DMSO was mixed and the mixture was sonicated using a Sonifier^®^ 450 cell disrupter (Branson Ultrasonics Co., Danbury, CT, USA) at output 1 and 50% duty cycle, for three 10-second pulses. The suspension was centrifuged at 17,900 × *g* for 5 min. The 1 ml soluble layer was collected into a 1.5 ml tube and was frozen with liquid nitrogen then stored at −80 °C prior to the purification experiment.

The purification of sonicated soluble fractions was performed using Glutathione Sepharose^®^ 4B (GE Healthcare) as a resin following the manufacturer’s protocol. Resin (100 µl) and 1 ml soluble fractions were mixed in a 2 ml tube and incubated at 4 °C for 16 h at 25 rpm with a RT-30mini rotator (TAITEC, Saitama, Japan). The resin was centrifuged at 500 × *g* for 5 min and the supernatant was discarded. The resin was washed five times with 1 ml TBS buffer. To the bound proteins 100 µl elution buffer (50 mM Tris-Cl, 10 mM reduced glutathione, pH 8.0; Nacalai Tesque) were added and the solution was incubated at 4 °C for 20 min at 25 rpm. After incubation, the resins were centrifuged at 500 × *g* for 5 min and the eluted fraction was collected. The elution step was repeated three times. The resulting solution was frozen with liquid nitrogen and stored at −80 °C prior to the pull-down assay.

### Pull-down assay

The pull-down assay was performed as previously described^59^. An aliquot (40 µl) of the elution fractions of GST-fused proteins purified with GST-Sepharose was used for the pull-down assay with Streptavidin Sepharose^®^ High Performance beads (GE Healthcare) to immobilize 40 nmol Bio-Cm or Bio-Ctrl. After mixing overnight, the resins were washed three times with 200 µl TBS buffer, mixed with SDS loading buffer containing 0.002% bromophenol blue, 5% glycerol, and 0.1% sodium dodecyl sulfate (Nacalai Tesque), and then incubated at 95 °C for 10 min. After centrifugation at 17,900 × *g* for 1 min, the supernatants were subjected to SDS-PAGE using 10% polyacrylamide gel. The separated proteins were blotted onto a PVDF membrane and detected by western blot analysis using Anti-Glutathione S-transferase antibody (Wako) as a primary antibody and goat anti-IgG AP (Santa Cruz Biotechnology Inc., Dallas, TX, USA) as a secondary antibody. The colorimetric detection of alkaline phosphatase activity was performed using CDP-Star (Thermo Fisher Scientific) with the ChemiDoc™ Imaging System (BIO-RAD Laboratories, Tokyo, Japan).

### Homology search and alignment of amino acid sequences

Protein sequences were downloaded from the National Center for Biotechnology Information database (http://www.ncbi.nim.gov). We used the BLAST tool to search for homologous sequences and CLUSTALW to align and compare amino acid sequences^60,61^.

## Supplementary information

Supplementary Information

## References

[1] MacDonald ML (2006). Identifying off-target effects and hidden phenotypes of drugs in human cells. Nat. Chem. Biol..

[2] Campillos M, Kuhn M, Gavin A-C, Jensen LJ, Bork P (2008). Drug Target Identification Using Side-Effect Similarity. Science..

[3] Dickson M, Gagnon JP (2004). Key factors in the rising cost of new drug discovery and development. Nat. Rev. Drug Discov..

[4] Futamura Y, Muroi M, Osada H (2013). Target identification of small molecules based on chemical biology approaches. Mol. Biosyst..

[5] Stoughton RB, Friend SH (2005). How molecular profiling could revolutionize drug discovery. Nat. Rev. Drug Discov..

[6] Dubach JM (2017). Quantitating drug-target engagement in single cells *in vitro* and *in vivo Nat*. Chem. Biol..

[7] Wilson K (2017). Detecting drug-target binding in cells using fluorescence-activated cell sorting coupled with mass spectrometry analysis. Methods Appl. Fluoresc..

[8] Chen X (2016). Drug-target interaction prediction: Databases, web servers and computational models. Brief. Bioinform..

[9] Talbot NJ (2003). On the Trail of a Cereal Killer: Exploring the Biology of *Magnaporthe grisea*. Annu. Rev. Microbiol..

[10] Hamer EJ (1987). A Mechanism for Surface Attachment. Science..

[11] Wilson RA, Talbot NJ (2009). Investigating the biology of plant infection by *Magnaporthe oryzae*. Nat. Rev. Microbiol..

[12] Fernandez, J. & Orth, K. Rise of a Cereal Killer: The Biology of *Magnaporthe oryzae* Biotrophic Growth. *Trends Microbiol*. (2018). 10.1016/j.tim.2017.12.007 PMC6003838 29395728

[13] Oh Yeonyee, Donofrio Nicole, Pan Huaqin, Coughlan Sean, Brown Douglas E, Meng Shaowu, Mitchell Thomas, Dean Ralph A (2008). Transcriptome analysis reveals new insight into appressorium formation and function in the rice blast fungus Magnaporthe oryzae. Genome Biology.

[14] Soanes, D. M., Chakrabarti, A., Paszkiewicz, K. H., Dawe, A. L. & Talbot, N. J. Genome-wide transcriptional profiling of appressorium development by the rice blast fungus *Magnaporthe oryzae*. *PLoS Pathog*. **8**, (2012). 10.1371/journal.ppat.1002514 PMC3276559 22346750

[15] Ishii A (2013). A eukaryotic molecular target candidate of roxithromycin: fungal differentiation as a sensitive drug target analysis system. Biosci. Biotechnol. Biochem..

[16] Kamakura T (1999). cDNA subtractive cloning of genes expressed during early stage of appressorium formation by *Magnaporthe grisea*. Biosci. Biotechnol. Biochem..

[17] Taft RJ, Pheasant M, Mattick JS (2007). The relationship between non-protein-coding DNA and eukaryotic complexity. BioEssays.

[18] HOLT R (1967). THE BACTERIAL DEGRADATION OF CHLORAMPHENICOL. The Lancet.

[19] Moazed D, Noller HF (1987). Chloramphenicol, erythromycin, carbomycin and vernamycin B protect overlapping sites in the peptidyl transferase region of 23S ribosomal RNA. Biochimie.

[20] Schlunzen F (2001). Structural basis for the interaction of antibiotics with the peptidyl transferase centre in eubacteria. Nature.

[21] Ibrahim NG, Burke JP, Beattie DS (1974). The sensitivity of rat liver and yeast mitochondrial ribosomes to inhibitors of protein synthesis. J Biol Chem.

[22] Smith GP, Petrenko VA (1997). Phage display. Chem. Rev..

[23] Satow R, Chan T, Asashima M (2002). Molecular cloning and characterization of dullard: a novel gene required for neural development. Biochem Biophys Res Commun.

[24] Hong J, Sung J, Lee D, Reddy RH, Kim YJ (2014). Selective Dephosphorylation by SCP1 and PP2A in Phosphorylated Residues of SMAD2. Bull. Korean Chem. Soc..

[25] Hayata T, Ezura Y, Asashima M, Nishinakamura R, Noda M (2015). Dullard/Ctdnep1 regulates endochondral ossification via suppression of TGF-β signaling. J. Bone Miner. Res..

[26] Wang Y (2011). Functional characterization of a NEM1-like gene in *Magnaporthe oryzae*. Agricultural Sciences in China.

[27] Rho HS, Kang S, Lee YH (2001). *Agrobacterium tumefaciens*-mediated Transformation of the Plant Pathogenic Fungus, *Magnaporthe grisea*. Mol. Cells.

[28] Nesti E, Corson GM, McCleskey M, Oyer JA, Mandel G (2014). C-terminal domain small phosphatase 1 and MAP kinase reciprocally control REST stability and neuronal differentiation. Proc. Natl. Acad. Sci..

[29] Kloet DEA (2015). FOXO target gene CTDSP2 regulates cell cycle progression through Ras and p21(Cip1/Waf1). Biochem. J..

[30] Naderi M (2017). Two triacylglycerol pathway genes, CTDNEP1 and LPIN1, are down-regulated by hsa-miR-122-5p in hepatocytes. Arch. Iran. Med..

[31] Beniaminov AD (2016). Interaction of two tumor suppressors: Phosphatase CTDSPL and Rb protein. Mol. Biol..

[32] Zhao Y (2014). C-terminal domain (CTD) small phosphatase-like 2 modulates the canonical bone morphogenetic protein (BMP) signaling and mesenchymal differentiation via smad dephosphorylation. J. Biol. Chem..

[33] Eliakim-Raz N (2014). Efficacy and safety of chloramphenicol: Joining the revival of old antibiotics? Systematic review and meta-analysis of randomized controlled trials. J. Antimicrob. Chemother..

[34] Burroughs AM, Allen KN, Dunaway-Mariano D, Aravind L (2006). Evolutionary Genomics of the HAD Superfamily: Understanding the Structural Adaptations and Catalytic Diversity in a Superfamily of Phosphoesterases and Allied Enzymes. J. Mol. Biol..

[35] Zhang M (2010). Structural and functional analysis of the phosphoryl transfer reaction mediated by the human small C-terminal domain phosphatase, Scp1. Protein Sci..

[36] Corden JL (2013). RNA polymerase II C-terminal domain: Tethering transcription to transcript and template. Chem. Rev..

[37] Eick D, Geyer M (2013). The RNA polymerase II carboxy-terminal domain (CTD) code. Chem. Rev..

[38] Hausmann S, Shuman S (2002). Characterization of the CTD phosphatase Fcp1 from fission yeast. Preferential dephosphorylation of serine 2 versus serine 5. J. Biol. Chem..

[39] Lin PS, Marshall NF, Dahmus ME (2002). CTD phosphatase: Role in RNA polymerase II cycling and the regulation of transcript elongation. Prog. Nucleic Acid Res. Mol. Biol..

[40] Kershaw MJ, Talbot NJ (2009). Genome-wide functional analysis reveals that infection-associated fungal autophagy is necessary for rice blast disease. Proc. Natl. Acad. Sci. USA.

[41] Jones JDG, Dangl JL (2006). The plant immune system. Nature.

[42] Dodds PN, Rathjen JP (2010). Plant immunity: towards an integrated view of plant–pathogen interactions. Nat. Rev. Genet..

[43] Mirkes PE (1974). Polysomes, ribonucleic acid, and protein synthesis during germination of *Neurospora crassa* conidia. J. bacteriol..

[44] Osherov N, May GS (2001). The molecular mechanisms of conidial germination. FEMS Microbiol. Letters.

[45] Huttlin EL (2017). Architecture of the human interactome defines protein communities and disease networks. Nature.

[46] Wu Y, Mark Evers B, Zhou BP (2009). Small C-terminal domain phosphatase enhances snail activity through dephosphorylation. J. Biol. Chem..

[47] Yeo M, Lin PS, Dahmus ME, Gill GN (2003). A novel RNA polymerase II C-terminal domain phosphatase that preferentially dephosphorylates serine 5. J. Biol. Chem..

[48] Park G, Xue C, Zheng L, Lam S, Xu JR (2002). MST12 regulates infectious growth but not appressorium formation in the rice blast fungus *Magnaporthe grisea*. Mol Plant Microbe Interact.

[49] Zhang H (2014). Pleiotropic Function of the Putative Zinc-Finger Protein MoMsn2 in *Magnaporthe oryzae*. Mol. Plant-Microbe Interact..

[50] Siniossoglou S, Hurt EC, Pelham HRB (2000). Psr1p/Psr2p, two plasma membrane phosphatases with an essential DXDX(T/V) motif required for sodium stress response in yeast. J. Biol. Chem..

[51] Kaida D, Yashiroda H, Toh-e A, Kikuchi Y (2002). Yeast Whi2 and Psr1-phosphatase form a complex and regulate STRE-mediated gene expression. Genes to Cells.

[52] Harata K, Nishiuchi T, Kubo Y (2016). *Colletotrichum orbiculare* WHI2, a Yeast Stress-Response Regulator Homolog, Controls the Biotrophic Stage of Hemibiotrophic Infection Through TOR Signaling. Mol. Plant. Microbe. Interact..

[53] Turrà D, Segorbe D, Di Pietro A (2014). Protein Kinases in Plant-Pathogenic Fungi: Conserved Regulators of Infection. Annu. Rev. Phytopathol..

[54] Kuroki M (2017). Chitin-deacetylase activity induces appressorium differentiation in the rice blast fungus *Magnaporthe oryzae*. Sci. Rep..

[55] Staben C (1989). Use of a bacterial hygromycin B resistance gene as a dominant selectable marker in *Neurospora crassa* transformation. Fungal Genet. Newsl..

[56] Watanabe S (2007). Mode of action of Trichoderma asperellum SKT-1, a biocontrol agent against *Gibberella fujikuroi*. J. Pestic. Sci..

[57] Kimura M, Kamakura T, Zhou Tao Q, Kaneko I, Yamaguchi I (1994). Cloning of the blasticidin S deaminase gene (BSD) from *Aspergillus terreus* and its use as a selectable marker for S*chizosaccharomyces pombe* and *Pyricularia oryzae*. MGG Mol. Gen. Genet..

[58] Studier F. William (2005). Protein production by auto-induction in high-density shaking cultures. Protein Expression and Purification.

[59] Kusayanagi T (2012). The antitumor agent doxorubicin binds to Fanconi anemia group F protein. Bioorganic Med. Chem..

[60] Altschul SF, Gish W, Miller W, Myers EEWW, Lipman DJ (1990). Basic local alignment search tool. Journal of Molecular Biology.

[61] Thompson JD, Higgins DG, Gibson TJ (1994). CLUSTAL W: improving the sensitivity of progressive multiple sequence alignment through sequence weighting, position-specific gap penalties and weight matrix choice. Nucleic Acids Res.

