# Supplementary material for: Chloramphenicol inhibits eukaryotic Ser/Thr phosphatase and infection-specific cell differentiation in the rice blast fungus
Source: Sci Rep. 2019 Jun 26;9:9283. doi: 10.1038/s41598-019-41039-x (PMC6594944; doi:10.1038/s41598-019-41039-x)
Supplement: Supplementary file 1 — Supplementary Information [file 41598_2019_41039_MOESM1_ESM.pdf]

Chloramphenicol inhibits eukaryotic Ser/Thr phosphatase and infection-specific cell differentiation in the rice blast fungus

Akihito Nozaka[1], Ayaka Nishiwaki[1], Yuka Nagashima[1], Shogo Endo[1], Misa Kuroki[1], Masahiro Nakajima[1], Megumi Narukawa[2], Shinji Kamisuki[3], Takayuki Arazoe[1], Hayao Taguchi[1], Fumio Sugawara[1] & Takashi Kamakura[1]*

[1]Tokyo University of Science, Department of Applied Biological Science, Faculty of Science and Technology, 2641, Yamazaki, Noda, Chiba, 278-8510, Japan
[2]Osaka University, Research Institute for Microbial Diseases, Department of Molecular Microbiology, 3-1 Yamadaoka, Suita, Osaka 565-0871 Japan
[3]Azabu University, Department of Veterinary Science, Laboratory of Basic Education, 1-17-71 Fuchinobe, Chuo-ku, Sagamihara-shi, Kanagawa 252-5201 Japan
Correspondence and requests for materials should be addressed to T.K. (email: kamakura@rs.noda.tus.ac.jp)

Supplementary Table1 Primers used in this study.

Name	sequence (5' to 3')
MoDul-Up-Fwd-KpnI	CAGGGTACCACGGGGACCAAAGCATC
MoDul-Up-Rvs-KpnI	GTGCGGTACCTTGGGCTAGACTGGATC
MoDul-Down-Fwd-XbaI	CGGGGTCTAGACGAAGAAGTGACAAAAG
MoDul-Down-Rvs-XbaI	GGCGTCTAGAGCTTGCGTAGGATCG
pTEF-Fwd-PmeI	TATGTTTAAACTAGCAAACGGTGGTCAAAGGATG
pTEF-Rvs-BamHI	TTAGGATCCTTTGACGGTGATGTATGGAAG
MoDul-EX-Fwd-XhoI	CCTGGGATCCCCGGAATTCATGTCTCGCGACGCCG
MoDul-EX-Rvs-BamHI	TCGGCCGCTCGAGTCGACCGAGTGTAACATCCAAT
MoDUL-Fwd	CCCCGACTAGTATGTCTCGCGACGCCGACG
MoDUL-Rvs	CGCTCACTAGTTCAGAGTGTAACATCCAATACT
CTDSP1-Fwd	GATCCACTAGTATGGACAGCTCGGCCGTCA
CTDSP1-Rvs	CTAGAACTAGTCTAGCTCCCTGGCCGTGGC
CTDSP2-Fwd	GATCCACTAGTATGGAACACGGCTCCATCA
CTDSP2-Rvs	CTAGAACTAGTCTAAGGGGCCCGCAGCTGC
CTDNEP-Fwd	CCTGGGATCCATGATGCGGACGCAGTGTC
CTDNEP-Rvs	CCGGGGATCCTCACCAGAGCCGATGTTGGT
CTDSPL-Fwd	GCTCGGATCCATGGACGGCCCGGCCATC
CTDSPL-Rvs	GTTAGGATCCCTACCTATTGCAGAGTCTGTGCA
CTDSPL2-Fwd	CCTGGGATCCATGAGGCTGAGAACACGGAAAG
CTDSPL2-Rvs	CCGGGGATCCTTAATCTGGGGGCAGCAAATCATG
MoDul-mutDxDxT-Fwd	TTGAATCTCAACGAGACGCTGGTTCATAG
MoDul-mutDxDxT-Rvs	CAGCGTCTCGTTGAGATTCAAAACTAAGCAC
CTDSP1-mutDxDxT-Fwd	CATCAACCTGAACGAGACCCTGGTG
CTDSP1-mutDxDxt-Rvs	CCAGGGTCTCGTTCAGGTTGATGACCAC

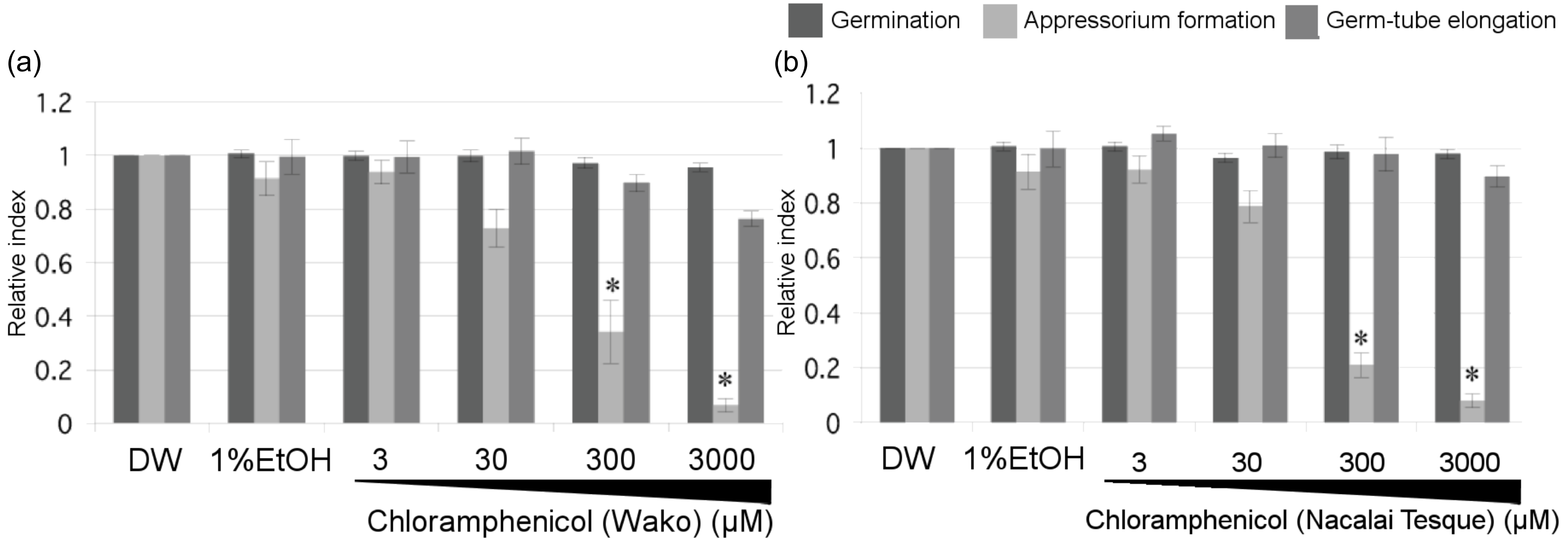

Supplementary Figure S1 Inhibitory effect of Cm on appressorium formation. Each conidial suspension of *M. oryzae* wild type P2 strain was inoculated with various concentrations of Cm purchased from **(a)** Wako **(b)** Nacalai Tesque diluted by 1% ethanol. The percentage of germination, germ-tube length and appressorium formation were assessed at 6 hpi. *$p < 0.05$ (Student's *t*-test) compared with 1% ethanol (control). The experiment was performed in triplicate for each sample and repeated three times.

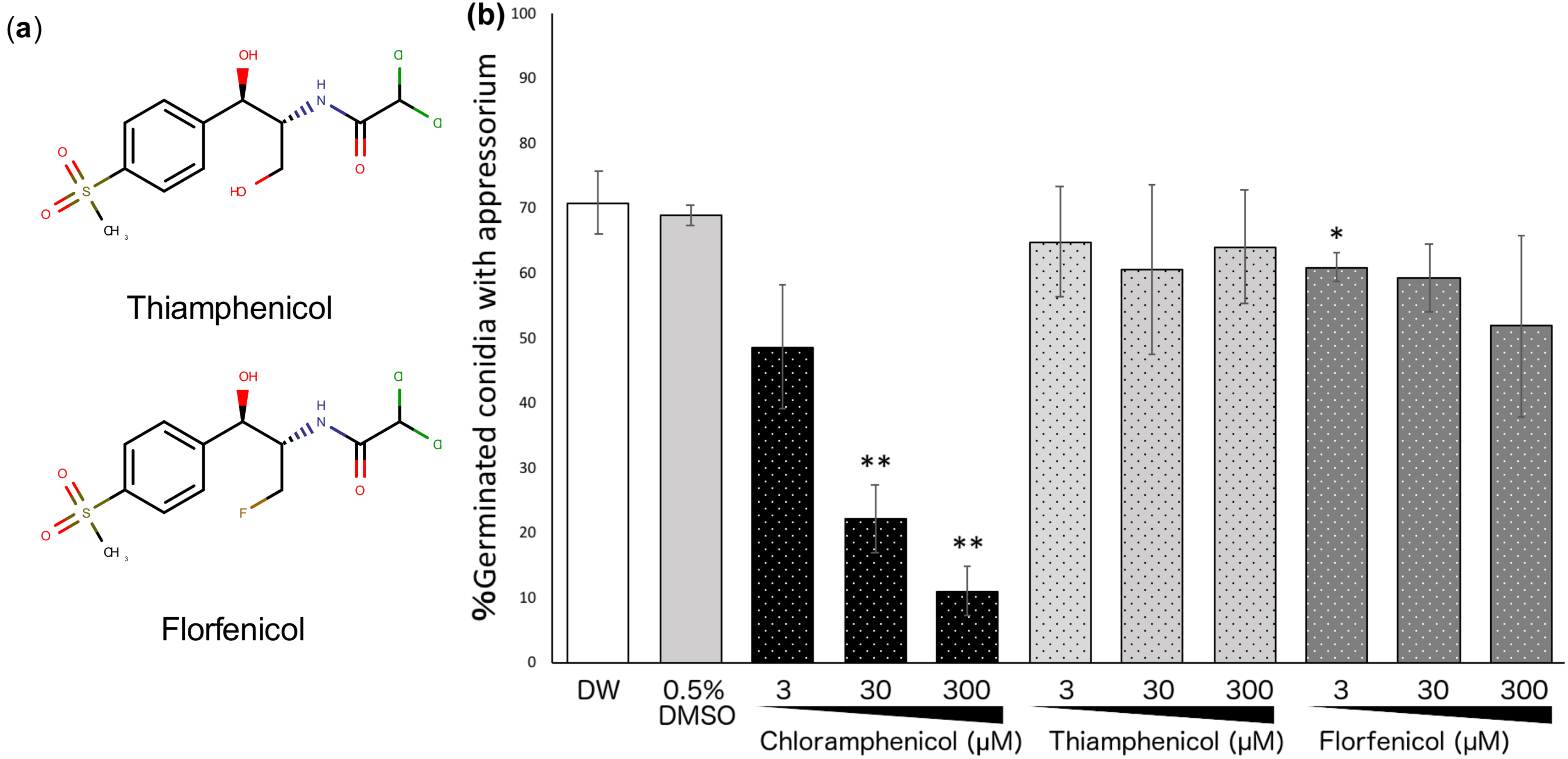

Supplementary Figure S2 Inhibitory effect of Cm analogs on appressorium formation. **(a)** Structures of thiamphenicol and florfenicol. **(b)** Appressorium formation percentage in each antibiotic. Each conidial suspension of *M. oryzae* wild type P2 strain was inoculated with various concentrations of Cm and analogs diluted by 0.5% DMSO. The percentage of appressorium formation was assessed at 6 hpi. *$p < 0.05$, **$p < 0.01$ (Student's *t*-test) compared with 0.5% DMSO (control). The experiment was performed in triplicate for each sample and repeated three times.

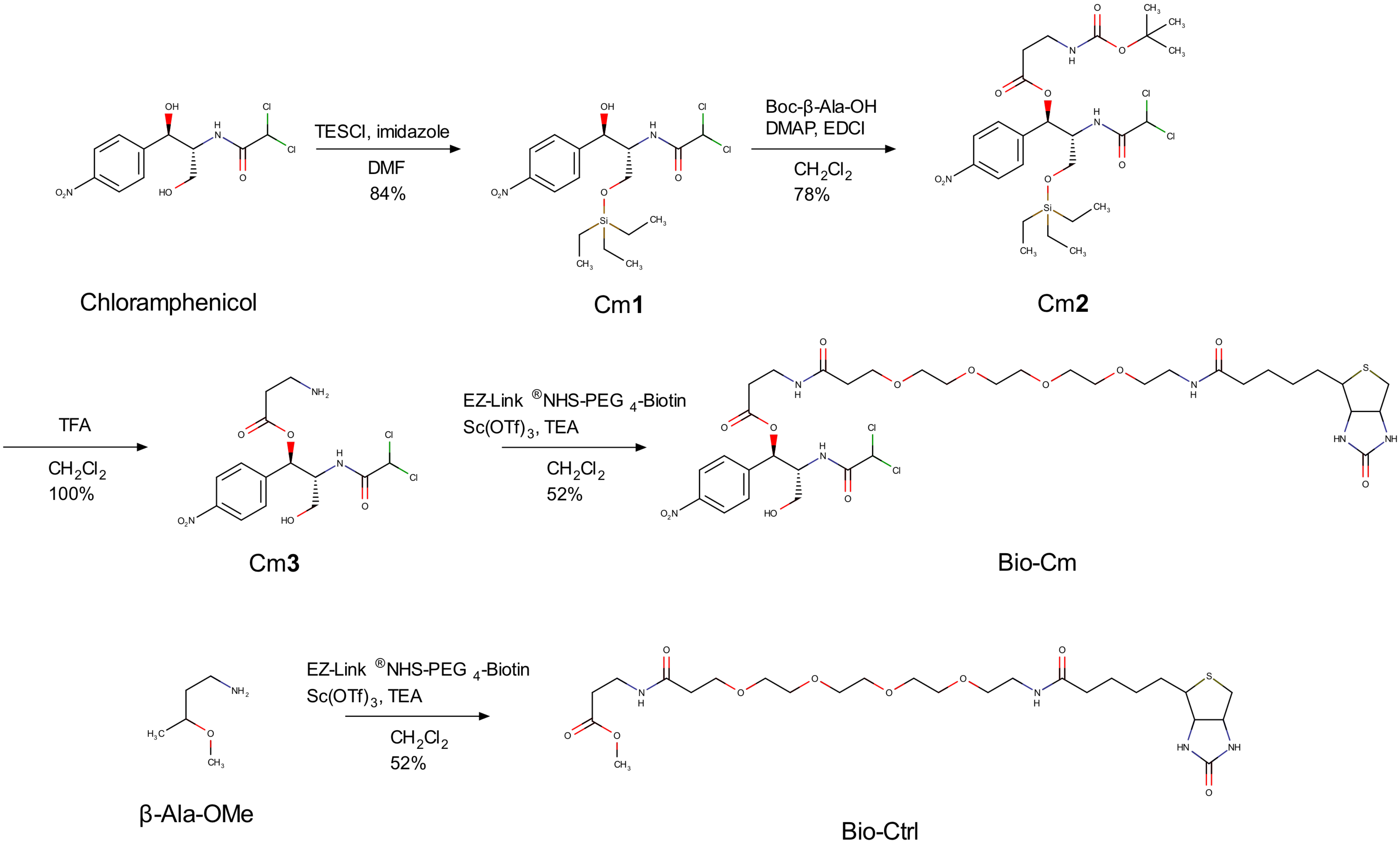

Supplementary Figure S3 Synthesis scheme of Bio-Cm and Bio-Ctrl

Supplementary Method1 Synthesis of Bio-Cm and Bio-Ctrl

The compounds produced by each reaction were checked by ^{1}H NMR spectra recorded at 600 MHz on an Avance DRX600 NMR spectrometer (Bruker, Karlsruhe, Germany). Biotinylated Cm was checked with an API QSTAR® Pulsar i ESI-MS spectrometer (AB SCIEX, Framingham, MA, USA). Flash column chromatography was performed on PSQ 100B silica gel (Fuji Silysia Chemical Ltd, Aichi, Japan).

Chloramphenicol (500 mg, 1.55 mmol; Wako) was dissolved in 15.5 mL *N*,*N*-dimethylformamide (Kanto Kasei Co., Ltd, Tokyo, Japan), imidazole (160 mg, 2.30 mmol) was added, and the solution was chilled at 0° C. Chlorotriethylsilane (300 µL, 1.70 mmol) (Kanto Kasei) was added and the mixture was stirred at room temperature for 45 min. The reaction was quenched with NH_4Cl (Wako), extracted with ethyl acetate (Wako), the organic layer was washed with saturated brine (Wako), dried with sodium sulfate, and evaporated *in vacuo*. The residue was purified by column chromatography using hexane (Wako) and ethyl acetate (hexane:ethyl acetate, 3:1 ; v/v) using silica gel to obtain Cm**1** (720 mg, 84%) .

Cm**1** (702 mg, 1.6 mmol) was dissolved in 16 mL CH_2Cl_2 (Wako) to which Boc-β-Ala-OH (607 mg, 3.2 mmol; Kanto Chemical Co., Inc., Tokyo, Japan), *N*,*N*-dimethyl-4-aminopyridine (392 mg, 3.2 mmol; Kanto Chemical), and 1-[3-(dimethylamino)propyl]-3-ethylcarbodiimide methiodide (615 mg, 3.2 mmol; Kanto Chemical) were added and stirred at room temperature overnight. The reaction mixture was poured into water and extracted with ethyl acetate. The organic layer was washed with brine, dried with sodium sulfate, and evaporated *in vacuo*. The residue was purified by column chromatography (hexane:ethyl acetate, 3:1) using silica gel to obtain 476.3 mg Cm**2** (476 mg, 78%).

Cm**2** (6.5 mg, 0.011 mmol) and 100 µL trifluoroacetic acid were mixed and stirred for 30 min. The reaction mixture was evaporated *in vacuo*, and the residue including Cm**3** was dissolved in 1.0 mL CH_2Cl_2. Scandium(III) triflate ($Sc(OTf)_3$; 1.0 mg, 2.1 µmol; Kanto Chemical) and 1 drop trimethylamine (Kanto Kasei) were added to the reaction mixture and left at −8° C. EZ-Link® NHS-PEG_4-Biotin (5.0 mg, 8.5 µmol; Pierce, Rockford, IL, USA) was dissolved in the reaction mixture and stirred at room temperature overnight. After incubation, the mixture was evaporated *in vacuo*. The residue was purified by column chromatography (ethyl acetate:methanol, 2:1 ; Wako) using silica gel to obtain Bio-Cm (2.0 mg, 52%). A positive ESI-MS fully supported the structure calculated for $C_{35}H_{52}Cl_2N_6O_{13}SNa$ [M + Na]+ 889.2690, found 889.2376.

Boc-β-Ala-OH (Kanto Chemical) was dissolved in 300 µL CH_2Cl_2, then $Sc(OTf)_3$ (2.0 mg, 3.4 µmol) and 1 drop trimethylamine were added to the reaction mixture and left at −8° C. EZ-Link® NHS-PEG_4-Biotin (5.0 mg, 8.5 µmol) was dissolved in the reaction mixture and stirred at room temperature for 60 min. After incubation, the mixture was evaporated *in vacuo*. The residue was purified by column chromatography (ethyl acetate:methanol, 2:1) using silica gel to obtain Bio-Ctrl (1.9 mg, 52%).

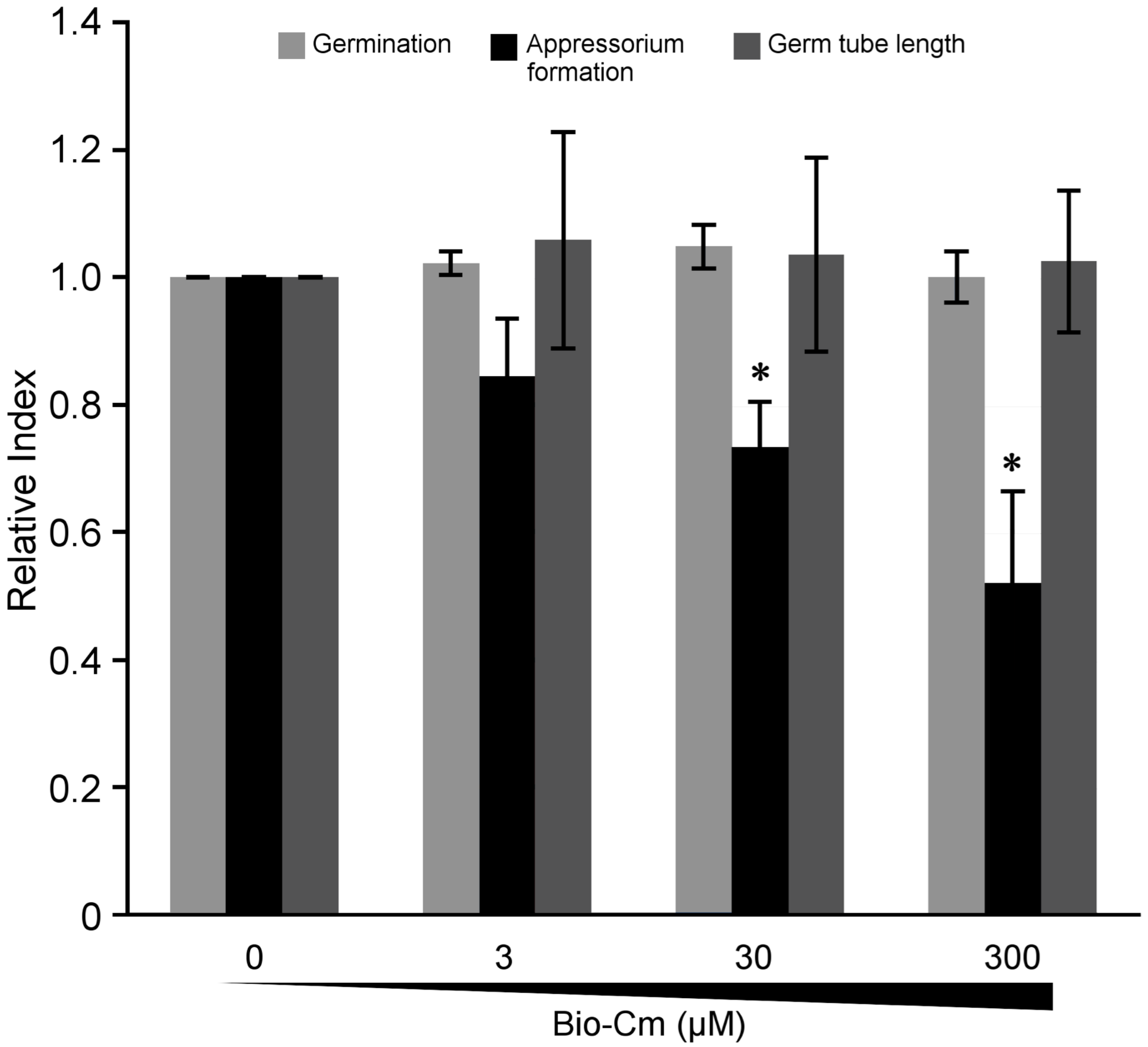

Supplementary Figure S4 Inhibitory effect of Cm connected to a biotinyl linker (Bio-Cm) on appressorium formation. Conidial suspensions of *M. oryzae* P2 were inoculated with various concentrations of Bio-Cm diluted with 1% ethanol. The percentage of germination, germ-tube length and appressorium formation were assessed at 6 hpi. *$p < 0.05$ (Student's *t*-test) compared with 0 μM Bio-Cm (control). The experiment was performed in triplicate for each sample and repeated three times.

Supplementary Table S2a Peptide sequences displayed by candidate phages and homology of the peptides

sample No.	peptide sequence	protein	e-value
P1	DQLQCWTIRNGDALALTSSHPMFPLGGRGAGVVRNPGSA	hypothetical protein (MGG_10792.7)	4.35266
P2	DLKDTIIIDNSPTSYIFHPQHAVPIRNLHSKGSKTVSPRETKTGRITVTGSKLPALPLRFETT	serine/threonine-protein phosphatase dullard (MGG_03646.7)	1.60E-09
P3	IRDIPEASEFCTEYMHPSSAFLQSPFFSKD	chitinase 1 (MGG_08054.7)	8.17666
P4	GSPFPGFYNQNILQVLFSEHLPMQRVYYRGWLKERYTGTCRSIKPG XTLYFSCLIHKASHLXEAPWYS	hypothetical protein (MGG_06624.7)	11.8701
P5	IHHPGNGLSGDRVQRSEEPSTSVGSGAPSTSLENPREGIILTPGRTH NRSRSPR	hypothetical protein (MGG_05643.7)	7.83517
P6	DLSFSFHSTQLTTSFYRSFPFXMRNPRSSRKAVXSDDGQTSLQR	hypothetical protein (MGG_04163.7)	40.9192
P7	GSEFQSEHLLQICSAPTHERLESPGRTRPLPSSRLSPTPTVRSNSEPPCHPQS	no candidate	—
P8	GSMKERLIPLTVINYPVSWSKQEENTGLPKPP	puromycin resistance protein pur8 (MGG_06794.7)	78.7352
P9	GSGLVASLMDINAWSSPQHSSGWAPGDG	pre-mRNA-splicing factor RSE1 (MGG_06457.7)	16.6961
P10	DPVPGQQGRAEPGQARPVAQDQSKNTL	hypothetical protein (MGG_04006.7)	45.9289
P11	DLCLDKETPRQGKGRGIKKKNYSRINQHT	C_2H_2 type zinc finger domain-containing protein (MGG_06328.7)	97.6818
P12	DHPSGSQGRATAGDALTKSTGDVSVIPKELVDIALHK	hypothetical protein (MGG_02116.7)	28.8247
P13	IITELKNSNPCEHNLCRCFGGHARRRFKTLIFSSISEPERQIIKTFNNGSKFSCREYPG	hypothetical protein (MGG_00804.7)	25.5951
P14	GSVLHEVMEQQTVSVAKAGIITTLNARTSILASANPIGSRYNPDRLLVRKGPP	DNA replication licensing factor mcm4 (MGG_09293.7)	1.10E-18
P15	IIRNVKEDESLSCADVPVSDALANHVGEKLSRKRPLRDKAAI	hypothetical protein (MGG_10275.7)	11.5671

Supplementary Table S2b Result of single clone check

sample No.	P/N ratio ([No. of phage binding to Bio-Cm]/[No. of phage binding to Bio-Ctrl])
P2	1.7
P14	0.8
empty phage 10-3b (Negative control)	1.1

Supplementary Method2 single clone check

NeutrAvidin™ Coated Plates, Clear, 8-Well Strip (Pierce) was washed with 200 µL TBS (10 mM Tris-HCl, pH 8.0, 0.8% NaCl) buffer in room temperature for 15 min on micro plate mixer MPX-96 (SCINICS, Tokyo, Japan). The buffer was removed and 200 µL biotinylated compound (0.5 nmol Bio-Cm or Bio-Ctrl solved in 10%DMSO in TBS buffer) was injected into the each well and immobilized for 60 min in room temperature. The wells were washed three times with 200 µL TBS buffer. Each phage clone (titer : 1.0×10^9 pfu/mL) 100 µL were injected into the wells and incubated for 60 min in room temperature. After incubation, supernatant was removed from wells and wells were washed 5 times with 200 µL TBS buffer. The bound phages were recovered by applying a 100 µL drop of host *Escherichia coli* (BLT5615) (OD_{600} = 0.5) and incubation for 15 min in room temperature. An aliquot (10 µL) was serial diluted with TBS buffer, then each 10 µL phage diluted solution were mixed with 500 µL *E. coli.* The mixture was inject to melted 3 mL LB liquid medium (37° C) and spread on LB plate and incubated until plaques were emerged. Positive/Negative ratio were calculated as the number of plaques of phage bound to Bio-Cm divided by the number of plaques of phage bound to Bio-Ctrl.

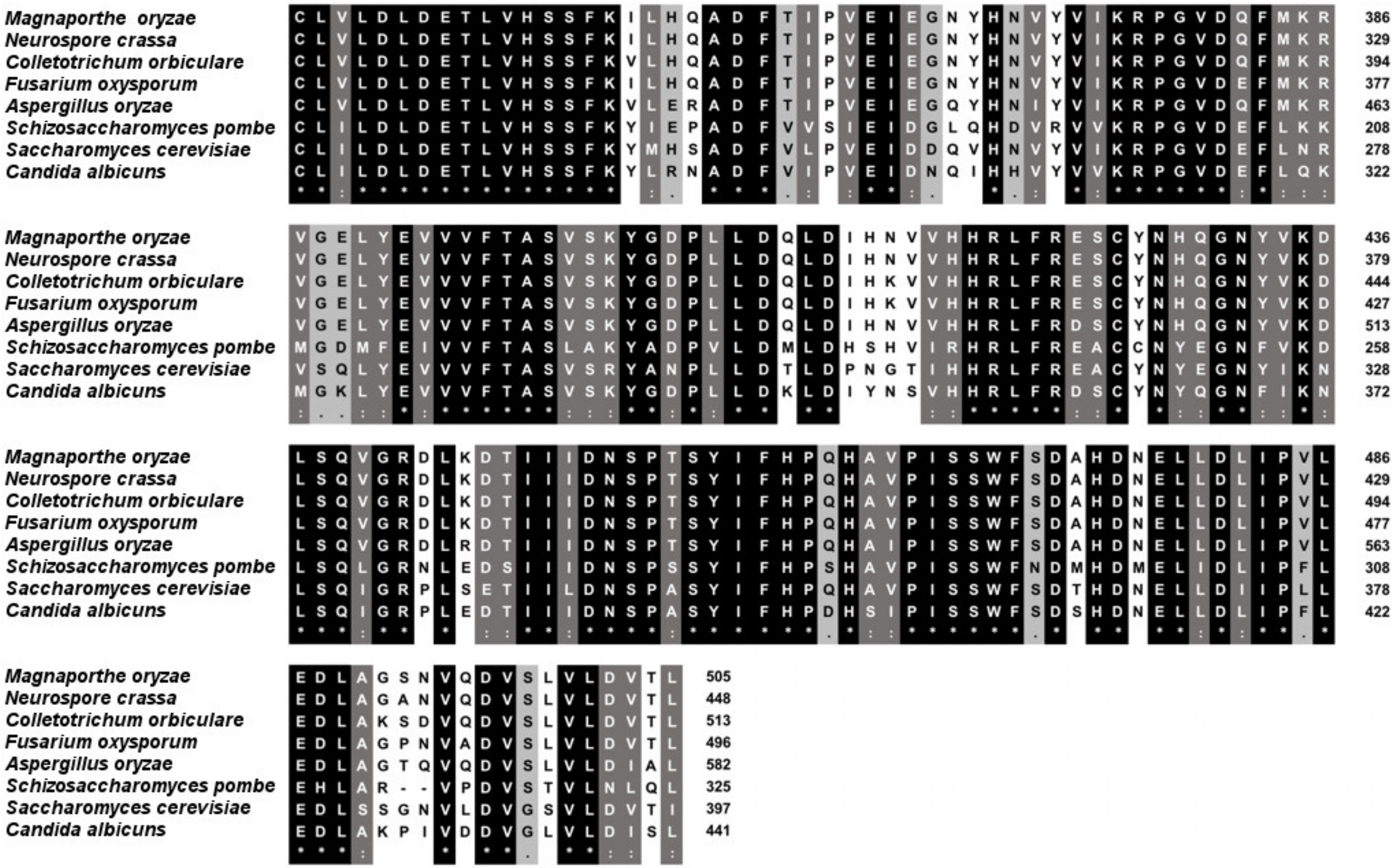

Supplementary Figure S5 Multiple alignment of Dullard phosphatase domain of MoDullard and orthologues in fungi. The Dullard domain sequences were derived from *Magnaporthe oryzae* (XP_003716243.1), *Neurospore crassa* (XP_011393200.1), *Colletotorichum orbiculare* (ENH79657.1), *Fusarium oxysporum* (EMT64576.1), *Aspergillus oryzae* (OOO10115), *Schizosaccharomyces pombe* (SPAC2F7.02c), *Saccharomyces cerevisiae* (AJV51493.1), and *Candida albicans* (XP_717778.1) . White letters on a black background indicate residues fully conserved in all sequences. White letters on a dark gray background indicate residues that belong to a strongly similar group. Black letters on a blight gray background indicate residues that belong to a weak similar group.

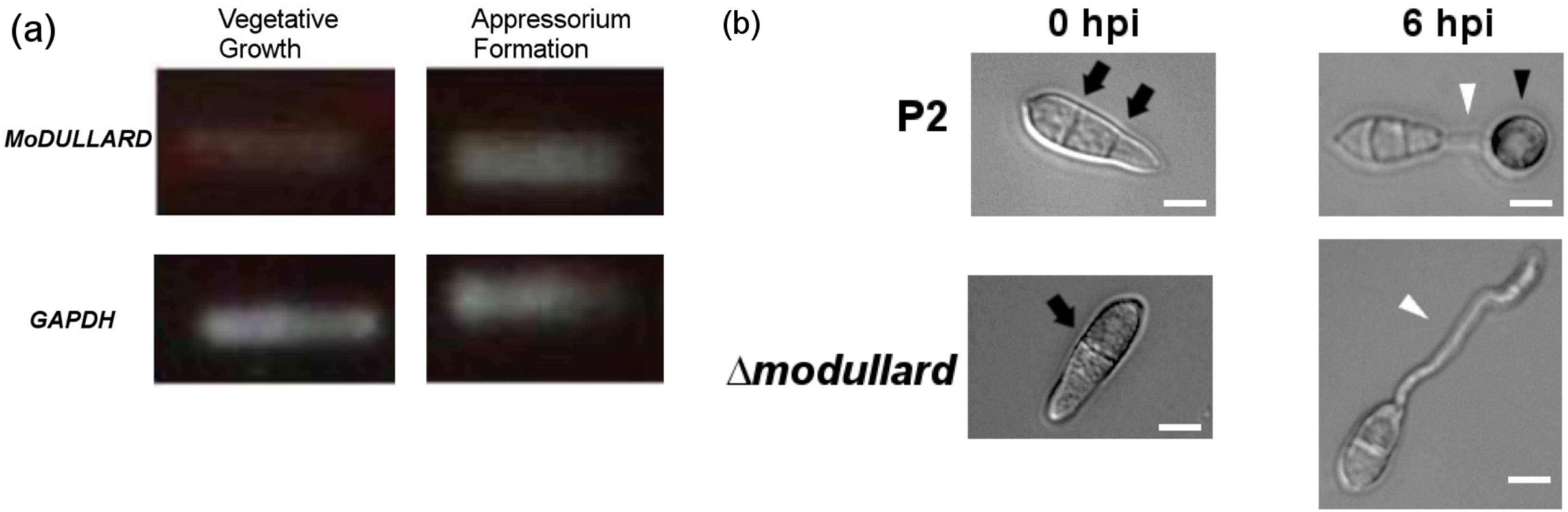

Supplementary Figure S6 (**a**) Semi-quantitative RT-PCR analysis of *MoDULLARD* expression. RNA was extracted at the vegetative growth phase and appressorium formation phase from wild-type *M. oryzae*. Expression of *GAPDH* was used as an internal control. (**b**) Conidia germinating on a hydrophobic surface (0 or 6 hours post infection; hpi). Black arrows indicate the septum in the conidium, white arrow heads indicate an elongated germ tube, and the black arrow head indicates an appressorium formed at the tip of a germ tube. Scales bar = 10 μm.

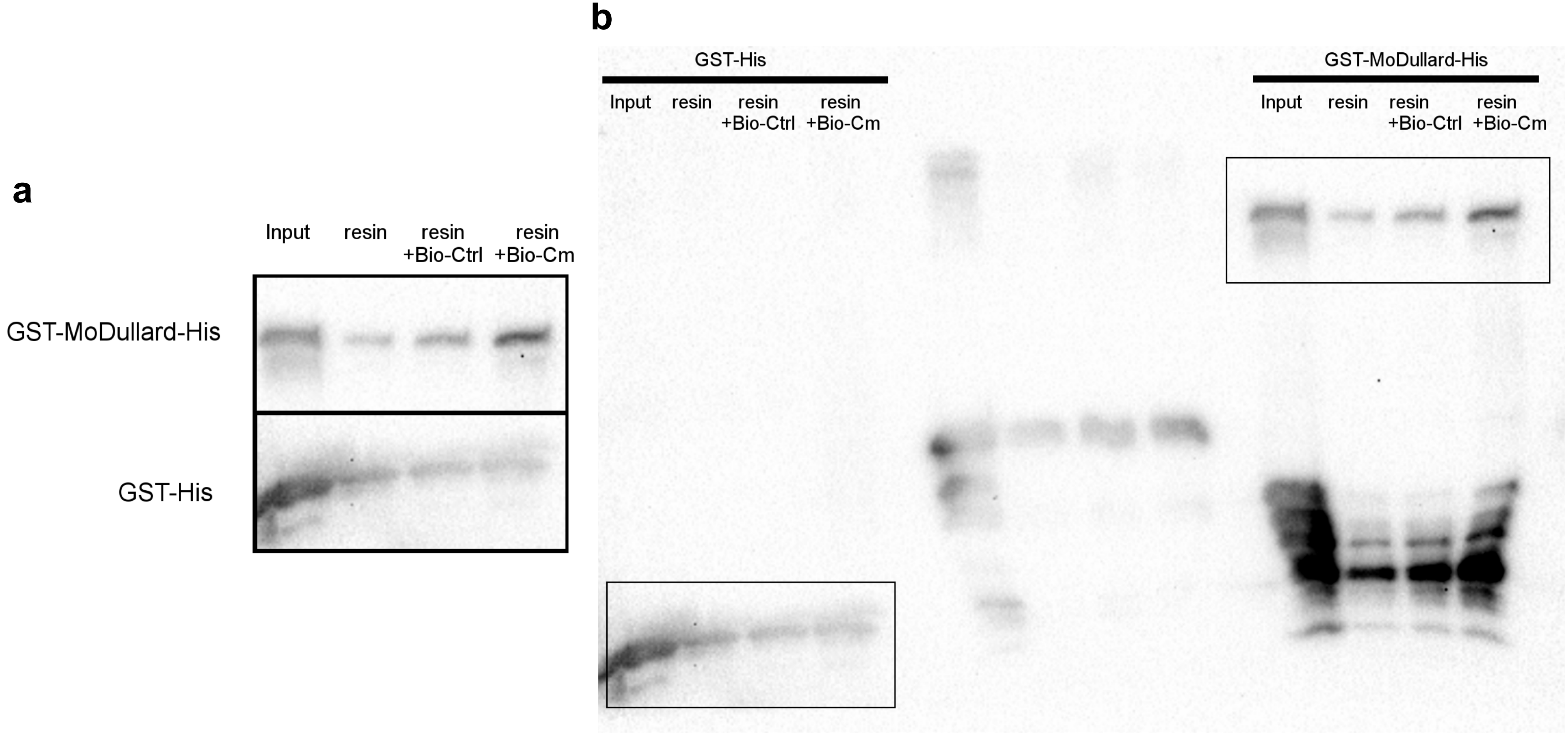

Supplementary Figure S7 (a) Detection of MoDullard binding to Cm using a pull-down assay. Cell extracts of *E. coli* over-expressing GST-MoDullard-His and GST-His (negative control GST-tag protein) were prepared and used for the assay. Proteins bound to only avidin sepharose resin, Bio-Ctrl immobilized resin, and Bio-Cm immobilized resin were isolated. Each protein was detected by western blot analysis using an anti-GST antibody. (**b**) Uncropped full-length blot.

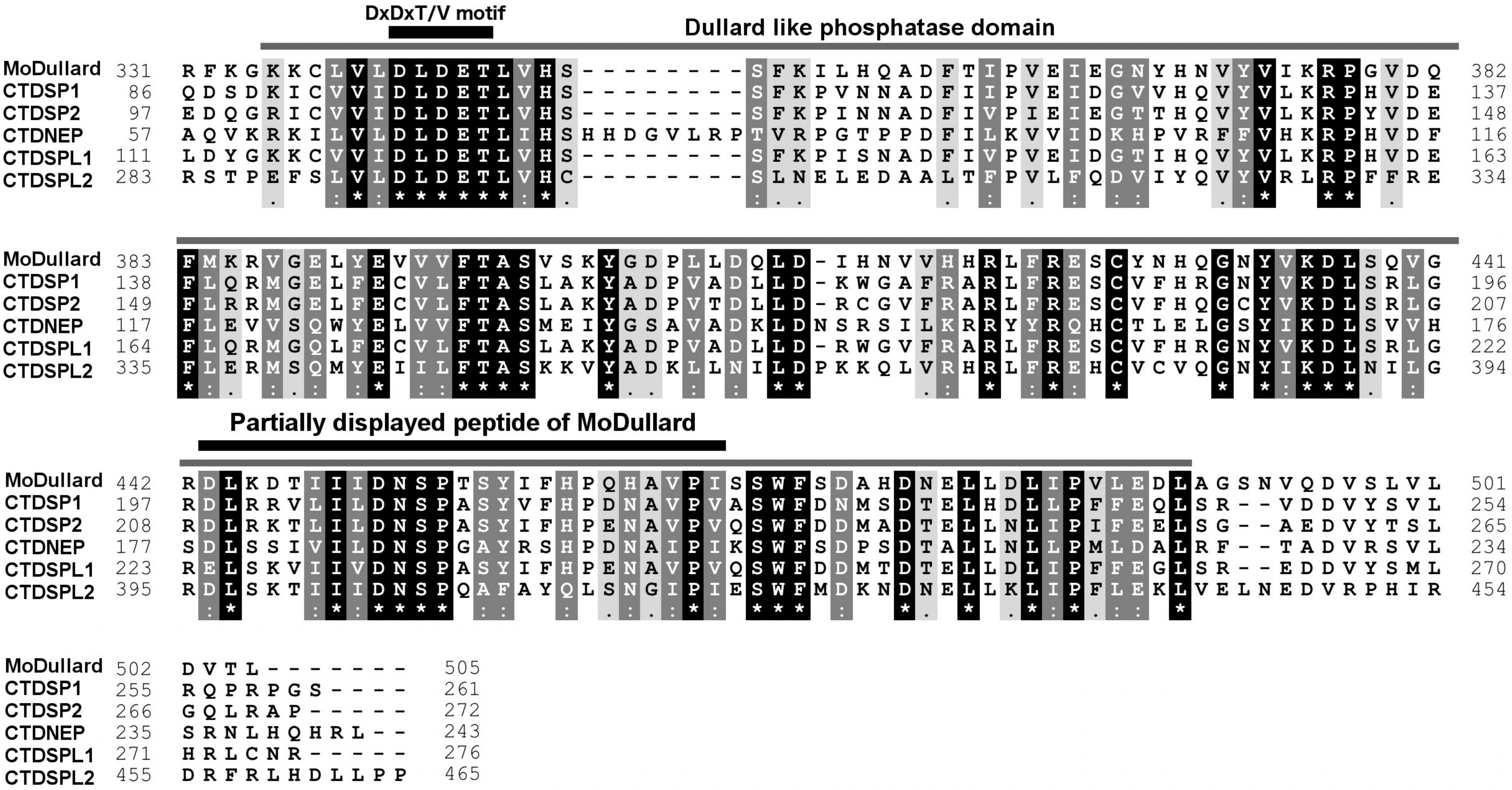

Supplementary Figure S8 Multiple alignment of Dullard phosphatase domain of MoDullard and orthologues in humans. White letters on a black background indicate residues fully conserved in all of the sequences. White letters on a dark gray background indicate residues that belong to a strongly similar group. Black letters on a blight gray background indicate residues that belong to a weak similar group. The phosphatase active site (DxDxT/V motif) and estimated chloramphenicol (Cm) binding site were highly conserved in all sequences.

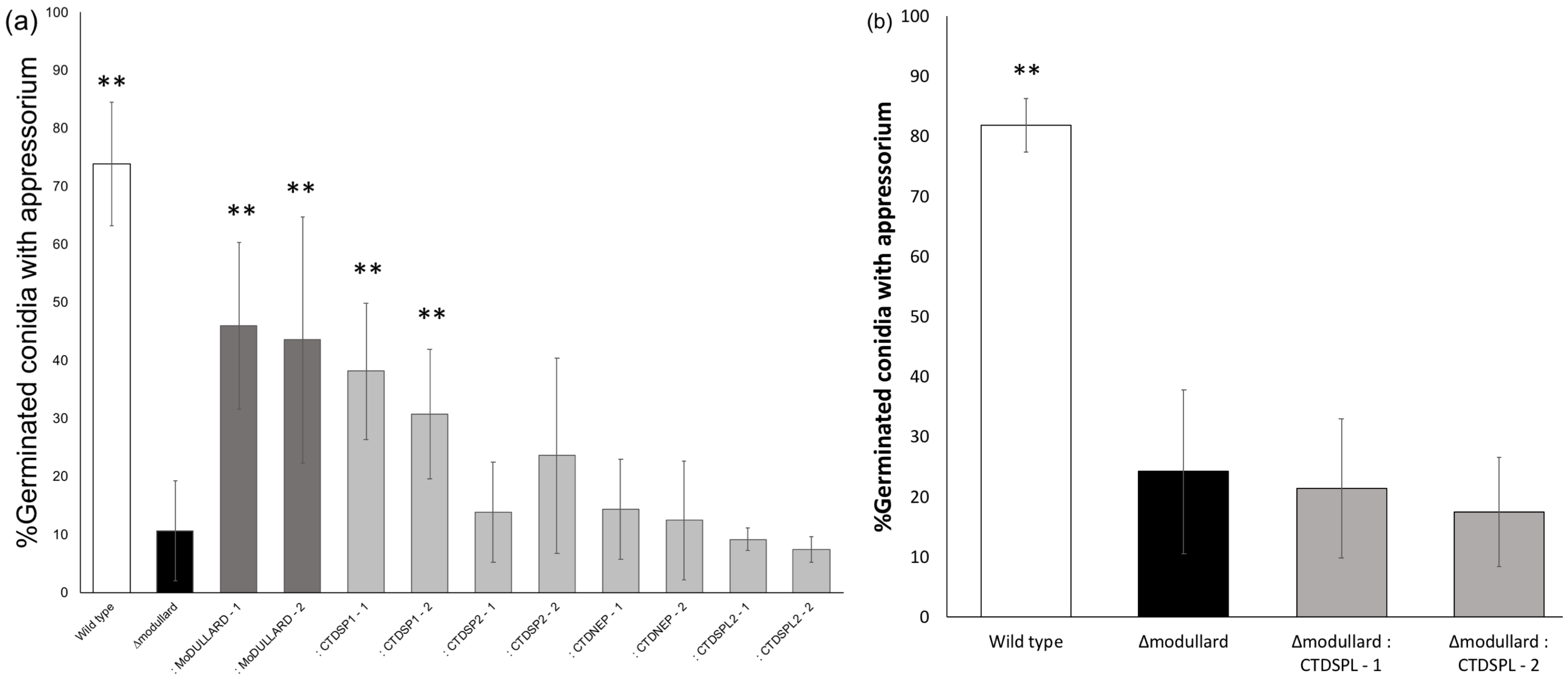

Supplementary Figure S9 Appressorium formation percentage in human homologue complementary strains. Each conidial suspension was treated with distilled water. (**a**) *MoDULLARD, CTDSP1, CTDSP2, CTDNEP* and *CTDSPL2* complementary strains and (**b**) *CTDSPL* complementary strains. Each conidial suspension was treated with distilled water. **p < 0.01 compared with Δ*modullard* (Student's *t*-test). Error bars indicate the standard error. These experiments were performed in triplicate for each sample and repeated three times.
